# Supplementary material for: Content-rich biological network constructed by mining PubMed abstracts
Source: BMC Bioinformatics. 2004 Oct 8;5:147. doi: 10.1186/1471-2105-5-147 (PMC528731; doi:10.1186/1471-2105-5-147)
Supplement: Additional File 2 — The original results of the above study (non-essential files are deleted to keep the file size under the limit set by BMC bioinformatics). [file 1471-2105-5-147-S2.bz2 › chilibotAdditionalFile2/dip05/695ID11722740E18020/html/TRAV_TRAK.html]

 


 **TRAV** and **TRAK** 
  
Found 3 abstracts in PubMed, retrieved 3.  
 

 What does Google say? 
 PDF only 
| .edu only 

---

**Interactive relationship** (e.g. stimulation, inhibition, etc)

**Neutral relationship**- Yeast two hybrid analysis with a  **traV**  bait repeatedly identified  **traK** , which is predicted to encode a periplasmic protein, among positive prey plasmids.  Ref: 11722740 Mol Microbiol, 2001
- A  **traK**  bait in turn identified  **traV**  and traB, which is predicted to encode an inner membrane protein.  Ref: 11722740 Mol Microbiol, 2001
- These results are consistent with a  **TraV**   **TraK**  TraB complex anchored to the outer membrane via the  **TraV**  lipoprotein.  Ref: 11722740 Mol Microbiol, 2001
- In contrast, TraB fractionated with the inner membrane in  **traV**  or  **traK**  mutant cells, and  **TraK**  appeared in the osmotic shock fluid from the  **traV**  mutant.  Ref: 11722740 Mol Microbiol, 2001
- Further, in  **traK**  mutant cells,  **TraV**  failed to accumulate to a detectable level, and the TraB level was significantly reduced, suggesting that  **TraV**  and TraB must interact with  **TraK**  for either protein to accumulate to its normal level.  Ref: 11722740 Mol Microbiol, 2001

**Non-interactive relationship** (e.g. studied together, co-existance, homology, etc.)

- Evidence that F plasmid proteins  **TraV** ,  **TraK**  and TraB assemble into an envelope spanning structure in Escherichia coli.  Ref: 11722740 Mol Microbiol, 2001
- Mutant analysis demonstrated that this synthesis is independent of host expression of traA, traL, traE,  **traK** , traB,  **traV** , traW, traC, traU, traF, or traH, but dependent on expression of the traF traH region.  Ref: 6219273 Mol Gen Genet, 1982
- Several additional observations support the hypothesis that  **TraV** ,  **TraK**  and TraB form a complex in Escherichia coli that spans the cell envelope from the outer membrane  **TraV**  through the periplasm  **TraK**  to the inner membrane TraB.  Ref: 11722740 Mol Microbiol, 2001
- First, two hybrid analyses indicated that  **TraV**  and TraB bind to different  **TraK**  segments, as required if  **TraK**  bridges a ternary complex.  Ref: 11722740 Mol Microbiol, 2001
- A polypeptide which migrate in the F pilin position was still present in membranes prepared from Flac strains carrying mutations in traL, traE,  **traK** , traB,  **traV** , traW, traC, traU, traF, traH or traG despite the inability of these mutants to elaborate F pili filaments.  Ref: 6120442 Mol Gen Genet, 1981
- Both  **TraK**  and  **TraV**  accumulated in traB2 Am cells.  Ref: 11722740 Mol Microbiol, 2001
